# Beyond Cholesterol Lowering: Clinical Caution, Personalization, and Nutritional Integration in Statin Therapy

**DOI:** 10.3390/nu18050722

**Published:** 2026-02-24

**Authors:** Giovanni Corsetti, Evasio Pasini

**Affiliations:** 1 Department of Clinical and Experimental Sciences, Università di Brescia, 25128 Brescia, Italy; evpasini@gmail.com; 2 Institute of Human Health, Ruaha Catholic University, Iringa P.O. Box 774, Tanzania

**Keywords:** statins, cardiovascular disease, nutritional status, malnutrition, amino acids, statin-associated muscle symptoms, sarcopenia, primary prevention

## Abstract

Background: Elevated low-density lipoprotein cholesterol (LDL-C) is a major risk factor for atherosclerosis and cardiovascular disease (CVD). Statins are the cornerstone of LDL-C reduction and are highly effective in secondary prevention. However, their benefit in primary prevention among individuals at low-to-moderate cardiovascular risk remains controversial, and long-term adherence is often limited by adverse effects. Methods: This narrative review summarizes current evidence on the clinical effectiveness of statin therapy, with particular attention paid to the role of nutritional status in modulating statin efficacy, safety, and interpretation of clinical outcomes. Results: In primary prevention the effectiveness of statins in reducing cardiovascular events remains mixed. Furthermore, 20–30% of patients in secondary or high-risk prevention do not achieve clinically meaningful benefits despite adequate LDL-C lowering. More than half of statin-treated patients discontinue therapy within two years, most commonly because of adverse effects, without a corresponding increase in cardiovascular mortality. Emerging evidence suggests that malnutrition and sarcopenia may significantly influence statin pharmacokinetics and pharmacodynamics, thereby affecting both therapeutic response and susceptibility to adverse events. In addition, statin-induced lipid lowering may alter nutrition-related biomarkers, potentially leading to misclassification or overestimation of malnutrition. Conclusions: Although statins remain effective agents for lowering LDL-C, their prescription should be embedded within an individualized, patient-centered approach. Current guidelines provide a robust methodological framework for statin use; however, their application should be contextualized rather than automatic. Optimal effectiveness is achieved when pharmacological therapy is integrated with dietary patterns, nutritional status, and lifestyle factors. Incorporating nutritional assessment into statin management may improve tolerability, enhance clinical outcomes, and enable more accurate cardiovascular risk stratification beyond standardized cholesterol-lowering strategies.

## 1. Introduction

Excess low-density lipoprotein cholesterol (LDL-C) is considered a key risk factor in the development of atherosclerosis responsible for cardiovascular (CV) diseases (CVD), and its reduction has been a therapeutic priority for decades. In this context, the 3-hydroxy-3-methyglutaryl coenzyme-A (HMG-CoA) reductase inhibitors are a class of drugs, also known as “statins”, that have revolutionized cardiovascular prevention, demonstrating significant reductions in morbidity and mortality from coronary and cerebrovascular events, which are the leading cause of morbidity and mortality globally, substantially contributing to the socioeconomic burden of healthcare systems [1].

Beyond lipid lowering, several clinical and mechanistic studies have described so-called pleiotropic effects of statins—including improvements in endothelial function, anti-inflammatory actions, atherosclerotic plaque stabilization and antithrombotic effects—which may contribute to early and some LDL-independent benefits observed in specific settings [2,3]. Nevertheless, evidence from randomized trials indicates that the magnitude of LDL-C reduction remains the primary determinant of clinical benefit, while the incremental clinical importance of pleiotropic mechanisms continues to be actively investigated [2,4].

Indeed, meta-analyses of statin trials have demonstrated that each 1 mmol/L reduction in LDL-C corresponds to an approximate 23% reduction in major vascular events [5]. For this reason, clinical guidelines worldwide strongly recommend statin for patients at CV risk, and their chronic use has become standard practice in both primary and secondary prevention strategies [6,7]. However, evidence on the effectiveness of statins in primary prevention remains mixed. Several studies suggest that, among low- to moderate-risk individuals—who represent approximately 60–70% of primary prevention populations—statin therapy may not be associated with a clearly demonstrable clinical benefit. This variability in findings may, at least in part, reflect differences in baseline cardiovascular risk across study populations [8]. In secondary or high-risk prevention, 20–30% of patients fail to achieve a clinically meaningful reduction in CV events despite adequate LDL-C reduction [9]. This suggests that CV risk is governed by a complex interplay of factors—including chronic inflammation, insulin resistance, and remnant cholesterol—some of which remain underestimated or insufficiently addressed in clinical practice. Nevertheless, despite the established therapeutic value of statins, many patients are forced to discontinue or down-titrate therapy, particularly because of muscular, hepatic, or metabolic adverse effects [10,11].

Multiple studies indicate that musculoskeletal adverse effects represent a primary determinant of both statin discontinuation and reluctance to initiate therapy. In an analysis of online health forum posts from more than 2000 statin users, approximately 90% reported discontinuing statin treatment due to musculoskeletal side effects [12]. Consistent with these findings, data from the PALM (Patient and Provider Assessment of Lipid Management) registry, which included 5693 adults, demonstrated that 55% of patients who discontinued statin therapy identified musculoskeletal symptoms as the primary reason. Among the 10% of patients who were reluctant to initiate statin treatment, concerns about adverse effects—most notably musculoskeletal symptoms, liver disease, and memory loss—were the most commonly reported barriers. Additional reasons included a preference for lifestyle modification, skepticism regarding the clinical benefits of statins, reluctance to use pharmacological therapies, a preference for natural remedies, and cost- or insurance-related considerations [13]. The Statin Adverse Treatment Experience survey, which included 1500 statin-treated patients, reported musculoskeletal symptoms in 22.1% of respondents, including muscle pain, cramps, stiffness, joint pain, muscle weakness, and fatigue [14]. Importantly, patient-reported musculoskeletal symptoms do not appear to be strictly dose-dependent. An observational real-world study of 1000 patients, 78% of whom were receiving low-intensity statin therapy, found that 10% reported musculoskeletal symptoms, demonstrating that such adverse effects can occur even at low statin doses [15].

A retrospective cohort study of 411,956 patients on statin therapy, designed to evaluate adherence and the difference between new and previously treated patients, showed that only 73% of new patients were adherent to therapy compared to 83.05% of those previously treated with statins. Persistence on treatment decreased to 92.65% after 3 months and 78.28% at 12 months [16]. More recently, it was estimated that over 50% of patients discontinue therapy within two years of initiation [17].

Globally, data from clinical trials and observational studies indicate that 20–25% of patients discontinue statins within 1–2 years, and that 5–10% experience true intolerance [18,19,20]. While some earlier research suggested no significant rise in CV mortality for statin discontinuers, more recent data show that stopping statins, especially in high-risk individuals, does significantly increase the risk of CV events and death [4,21]. However, discontinuation or suboptimal adherence to statin therapy remains a major clinical challenge, as it represents a missed opportunity to reduce the burden of CVD [22]. Consequently, the risk/benefit balance of statin use in primary prevention remains a complex and evolving topic of debate [23].

Within this context, nutritional status has emerged as a critical factor that may influence the pleiotropic effects of statins, thereby linking pharmacological mechanisms with diet-related and malnutrition-associated clinical outcomes.

Malnutrition is an increasingly prevalent clinical condition among patients with chronic diseases, particularly in CV and geriatric–metabolic settings characterized by inflammation and complex metabolic disturbances. In these populations, the prevalence of malnutrition ranges from 30% to 60% [24]. Recently, growing attention has been directed towards the interplay between nutritional status and statin therapy. Malnutrition and sarcopenia may alter drug metabolism, thereby affecting both the efficacy and tolerability of statins [25,26]. Moreover, chronic statin use, through marked reductions in total cholesterol and LDL-C levels, may interfere with nutrition-related indices that include lipid parameters, such as the CONUT score, potentially leading to overestimation of malnutrition [27,28]. In frail or elderly individuals, low LDL-C levels may not necessarily reflect CV protection, but rather indicate nutritional decline or chronic inflammation [29,30]. Taken together, these observations highlight the need for a deeper understanding of the bidirectional relationship between malnutrition and statin therapy, with particular attention paid to underlying pathophysiological mechanisms, clinical implications, and the interpretative limitations of current diagnostic tools.

Statin efficacy and risk of adverse effects, including muscle toxicity and new-onset diabetes, show significant interindividual variability influenced by genetic polymorphisms (e.g., SLCO1B1 and cytochrome P450 variants), demographic factors such as age and sex, and ethnic background, suggesting that pharmacogenomic profiling may enhance personalized statin therapy [31].

This narrative review aims to summarize the available evidence and to highlight how an integrated and personalized approach—incorporating nutritional assessment—may optimize clinical outcomes and therapeutic management in patients receiving statin therapy, beyond standardized cholesterol-lowering strategies.

Our aim is not to criticize or downplay the usefulness of statins, but rather to emphasize the importance of a personalized and integrated therapeutic approach in which nutrition plays a fundamental role in enhancing treatment efficacy and, above all, in reducing the harmful side effects of statins, particularly at the muscular level. To this end, a bibliographic search was conducted on the main scientific literature databases, focusing on the most recent studies, using keywords such as “statin”, “cardiovascular risk”, “statin-associated muscle symptoms”, “malnutrition”, and “adverse effect of statins”. To enhance transparency in all aspects of this narrative and qualitative research, we followed the recommendations of the Standards for Reporting Qualitative Research (SRQR) [32].

## 2. Statins: Mechanism of Action and Pharmacokinetic Aspects

The mechanism of action of statins and their pharmacokinetics has been extensively described in the literature. Therefore, this section provides a concise overview of the key aspects relevant to the present discussion.

**Figure 1:**
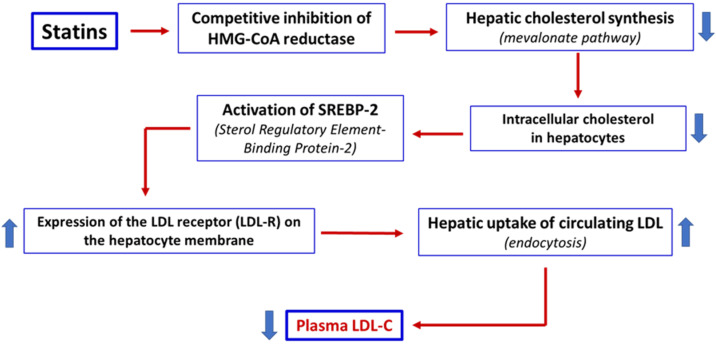
Statins act as competitive inhibitors of HMG-CoA reductase, a key enzyme in the mevalonate pathway, resulting in a reduction in endogenous cholesterol synthesis in the liver. The reduction in intracellular cholesterol activates the transcription factor SREBP-2, which induces the upregulation of LDL receptors (LDL-R) on the surface of hepatocytes. Increased LDL-R expression increases the clearance of LDL from the bloodstream, resulting in a reduction in plasma LDL-C levels. Thick arrows up = increase. Thick arrows down = decrease.

Statins are approximately 90% bound to serum proteins, mainly albumin, except for pravastatin, which exhibits a protein-binding rate of about 50% [33]. Hepatic uptake of statins is mediated by active transporters, particularly organic-anion-transporting polypeptide 1B1 (*OATP1B1*; *SLCO1B1* gene). Genetic variants (e.g., *SLCO1B1 c.521T>C*) or inhibitors of *OATP1B1* can increase plasma and skeletal muscle exposure to certain statins, thereby increasing the risk of muscle toxicity [34]. In the liver, statins act as competitive inhibitors of HMG-CoA reductase, blocking the conversion of HMG-CoA to mevalonate. This inhibition reduces endogenous hepatic cholesterol synthesis, upregulates the expression of LDL receptors on the hepatocytes’ surface, and consequently enhances the clearance of circulating LDL-C levels, leading to a reduction in plasma LDL-C levels [3] (Figure 1).

In addition to their lipid-lowering effects, statins exert a range of pleiotropic actions mediated by reduced synthesis of intermediates of the mevalonate pathway, including isoprenoids such as farnesyl pyrophosphate and geranyl-geranyl pyrophosphate. These intermediates regulate the activity of prenylated proteins (e.g., Rho and Ras), thereby influencing key biological processes such as inflammation, endothelial function, plaque stability, and coagulation. These mechanisms are particularly relevant when considering the CV benefits of statins that are independent of LDL-C reduction [3].

Statins are commonly classified as lipophilic or hydrophilic. Lipophilic statins (atorvastatin, simvastatin, lovastatin, fluvastatin, pitavastatin) more readily penetrate extrahepatic tissues and are metabolized by cytochrome P450 (CYP450) enzymes, particularly CYP3A4 and CYP2C9. In contrast, hydrophilic statins (rosuvastatin, pravastatin) are more hepato-selective and exhibit limited distribution in peripheral tissues. These differences influence pleiotropic effects, safety profiles (particularly the risk of myopathy) and susceptibility to drug–drug interactions [35]. Some statins (simvastatin, lovastatin) are administered as inactive lactone prodrugs and are converted by hepatic enzymes to the active β-hydroxy acid form, whereas others (pravastatin, rosuvastatin, atorvastatin) are administered directly in their active form. This distinction has implications for pharmacokinetics and potential drug interactions [3].

Although most statins are well absorbed following oral administration, their systemic bioavailability is limited by extensive first-pass hepatic metabolism. Therefore, for example, the bioavailability of atorvastatin is approximately 12%. In general, the oral bioavailability of statins ranges from 5 to 30%. The plasma half-life varies widely among the different molecules: from approximately 1–3 h for simvastatin and lovastatin, and up to or over 19 h for rosuvastatin and atorvastatin. This variability influences the dosage and therapeutic efficacy at the same dose [35].

Several statins (atorvastatin, simvastatin, and lovastatin) are primarily metabolized in the liver via the cytochrome CYP3A4 enzyme system and subsequently undergo conjugation with glucuronic acid by uridine 5′-diphospho-glucuronosyltransferase (UGT) enzymes. These statins are predominantly eliminated via biliary excretion. In contrast, hydrophilic statins such as pravastatin are less dependent on hepatic metabolism and are extensively eliminated through the kidneys.

## 3. Disadvantages of Chronic Statin Therapy

Statins are significantly used in managing hypercholesterolemia and have many pleiotropic effects. Although statins represent a well-established therapeutic strategy capable of reducing CV morbidity and mortality through synergistic mechanisms that extend beyond simple plasma LDL-C lowering, long-term adherence remains problematic [4,17]. Indeed, they also have many dose-dependent adverse effects that necessitate caution in their widespread use [4]. This issue is largely driven by perceived or actual adverse effects, which most commonly involve skeletal muscle [18,19,36].

A review based on evidence gathered in PubMed shows that, despite all of their beneficial effects, statins may affect mitochondrial function, and that some adverse effects might be mediated through mitochondrial dysfunction and intracellular surviving pathways [37]. For example, cardiomyocytes obtained from ten patients with metabolic syndrome (MetS) treated with simvastatin (MetS+Stat) were found to be more susceptible to stress both before and after ischemia–reperfusion during cardio-pulmonary bypass than cardiomyocytes from ten MetS patients not receiving statins. Specifically, cardiomyocytes from MetS+Stat patients exhibited reduced expression of the mitochondrial chaperone GRP75, together with increased expression of the endoplasmic reticulum stress marker GRP78 and the pro-apoptotic protein Bax [38]. In addition, statin therapy has been associated with a reduction in the omega-3-to-omega-6 fatty acid ratio, as well as with increases in body mass index, insulin resistance, and the risk of diabetes [39]. A recent systematic review of 11 articles with a total of 46,728,889 participants provides compelling evidence that statins are significantly associated with a decrease in insulin sensitivity, regardless of the type of statin used. Therefore, personalizing therapy by selecting statins with a lower risk of causing insulin intolerance can optimize treatment outcomes in patients at higher risk of developing type 2 diabetes [40].

Statins appear to have a neutral effect on the risk of cancer and cancer death in randomized clinical trials (RCTs) [41]. On the contrary, more recently, statins have been linked to reduced antitumor immune surveillance and a potential increase in breast cancer risk [39]. However, a review of 31 cohort studies involving 261,834 female breast cancer patients suggests statins may help reduce cancer recurrence and death rates [42]. In addition, a study of 67,768 Japanese participants found that longer duration of cholesterol-lowering drug use was significantly associated with a reduced risk of liver cancer and an increased risk of pancreatic cancer, with sex-specific differences observed in lung cancer risk [43]. In essence, to date, the correlation between statins and tumors is controversial and requires further studies.

The clinical effectiveness of statins may be further compromised by statin-associated muscle symptoms (SAMSs), which represent the most frequently reported adverse effect [11,18,19]. In contrast to evidence from RCTs, findings from patient registries and real-world clinical experience suggest that 7–29% of patients experience SAMSs, commonly associated with normal or mildly elevated creatine kinase (CK) levels [18]. SAMSs encompass a broad spectrum of manifestations, ranging from myalgia and muscle weakness to severe myalgia and elevations in CK levels [44]. Statin-induced myotoxicity includes a self-limiting toxic myopathy that resolves after statin discontinuation, as well as a rare immune-mediated condition—immune-mediated necrotizing myopathy (IMNM)—which requires immunomodulatory treatment [45].

It is important to highlight that SAMSs are partly determined by genetic factors affecting statin disposition, muscle vulnerability, and immune responses. Loss-of-function variants in SLCO1B1 and polymorphisms in statin-metabolizing enzymes (CYP3A4, CYP3A5, CYP2D6) and transporters (ABCB1, ABCG2) increase systemic statin exposure and SAMS risk. At the muscle level, statins may unmask latent myopathies linked to variants in CPT2, PYGM, RYR2, and LPIN1. Immune-related mechanisms include associations with LILRB5 variants and, in susceptible individuals carrying the HLA-DRB1*11:01 allele, the development of anti–HMG-CoA reductase autoantibodies, which may directly contribute to muscle injury [46,47]

The pathophysiology of SAMSs is multifactorial and remains incompletely understood. Several mechanisms have been proposed, including mitochondrial dysfunction [48,49], disruption of intracellular signaling pathways [50], vitamin D deficiency [51], inhibition of the mevalonate pathway [52], genetic susceptibility [47], and underlying neuromuscular disorders [53].

A central mechanism underlying statin-induced muscle toxicity is the inhibition of the mevalonate pathway. In addition to reducing cholesterol synthesis, this pathway also limits the production of isoprenoid intermediates such as coenzyme Q10 (CoQ10, also known as ubiquinone), farnesyl pyrophosphate, and geranylgeranyl pyrophosphate [37]. These compounds are essential for mitochondrial function, protein prenylation, and cellular energy metabolism. Reduced CoQ10 availability impairs the mitochondrial respiratory chain, leading to decreased adenosine triphosphate (ATP) production and increased generation of reactive oxygen species (ROS). The resulting oxidative stress promotes mitochondrial DNA damage and activation of apoptotic pathways, ultimately contributing to muscle fiber injury [37,54].

**Figure 2:**
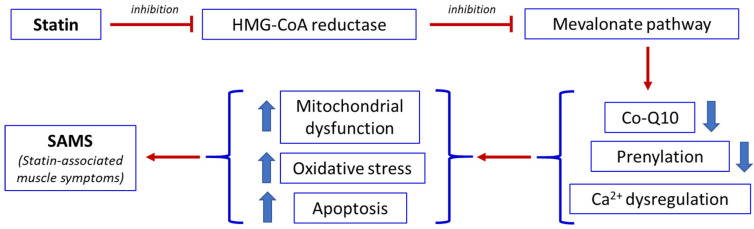
Simplified diagram of the main biochemical steps of the consequences of statin administration leading to statin-associated muscle symptoms (SAMS). Thick arrows up = increase. Thick arrows down = decrease.

Statins may also disrupt intracellular calcium homeostasis in skeletal muscle, contributing to symptoms such as cramps and spasms [55,56]. Furthermore, they can alter the expression of genes involved in muscle protein degradation and mitochondrial biogenesis, thereby promoting muscle atrophy and impairing regenerative capacity [55]. Finally, statins may additionally increase mitochondrial membrane permeability, facilitating the release of pro-apoptotic enzymes and further disturbing calcium handling [55]. In most cases, SAMSs are mild and reversible. However, a small subset of patients may develop severe complications, including rhabdomyolysis [57] and necrotizing autoimmune myopathy (NAM), which is characterized by muscle weakness, markedly elevated creatine kinase (CK) levels, and the presence of anti-HMG-CoA reductase antibodies [11]. In addition, the skeletal muscle of statin-treated rats showed an increase in slow myosin heavy-chain (MHC) isoforms, suggesting a shift from a fast to a slow muscle fiber phenotype [58]. Figure 2 summarizes the sequence of events that, following statin administration, leads to the development of SAMSs.

## 4. Limitations of Guidelines and Clinical Trials

International guidelines for the management of dyslipidemia play a central role in defining diagnostic criteria, therapeutic targets, and indications for statin prescription [59,60,61]. These guidelines are primarily based on evidence derived from RCTs and their systematic reviews and meta-analyses, which are widely regarded as the gold standard for evaluating interventions aimed at reducing cardiovascular risk through LDL-C lowering.

However, although RCTs represent the most rigorous method for evaluating the effectiveness and safety of healthcare interventions, their results must be interpreted considering important methodological, operational, and ethical limitations. Consequently, RCTs may fail to adequately reflect the heterogeneity of real-world patient populations, a limitation that has been increasingly recognized and addressed by regulatory authorities such as the U.S. Food and Drug Administration (FDA) [62,63].

To ensure internal validity, many RCTs adopt restrictive eligibility criteria that exclude specific patient groups, such as individuals with multimorbidity, advanced age, polypharmacy, or socioeconomic vulnerability [63,64]. These exclusions limit external validity and reduce the generalizability of trial findings to everyday clinical settings. Consequently, therapeutic strategies that demonstrate robust efficacy in selected trial populations may yield attenuated benefits in broader, unselected patient cohorts [56]. In addition, the under-representation of vulnerable populations limits meaningful subgroup analyses and hinders the extrapolation of results across diverse demographic and clinical contexts [65]. Another relevant limitation is that many RCTs prioritize short-term endpoints to reduce costs and accelerate trial completion, often resulting in insufficient follow-up to capture long-term benefits, delayed adverse effects, or disease progression [54]. This issue is particularly problematic for preventive therapies and chronic conditions, where clinically meaningful outcomes may emerge only over extended periods. Furthermore, publication and reporting biases must be considered, as trials with positive findings are more likely to be published and disseminated than those reporting neutral or unfavorable results [66,67].

Taken together, these considerations underscore the importance of interpreting clinical practice guidelines not as rigid prescribing algorithms but as flexible, evidence-informed frameworks that support personalized clinical decision-making. Such an approach is essential given inter-individual variability in statin response, the presence of comorbidities, patient preferences, and the complex interaction between pharmacological therapy and dietary habits.

Current guidelines appropriately emphasize the role of lifestyle modification, recognizing nutritional intervention as a fundamental component of dyslipidemia management, both as a first-line strategy and as an adjunct to pharmacological treatment [68,69]. For example, Mediterranean-style dietary patterns, characterized by a high intake of favorable nutrients—such as soluble fiber, mono- and polyunsaturated fatty acids, phytosterols, and antioxidants—have been shown to improve lipid profiles and enhance the lipid-lowering efficacy of statins [70]. Conversely, dietary patterns rich in saturated fats, refined sugars, and alcohol may attenuate therapeutic response and contribute to residual cardiovascular risk.

A further illustration of the need for context-sensitive application of guidelines concerns younger populations. Among children and adolescents, excess body weight resulting from poor dietary habits, high consumption of ultra-processed foods, and sedentary lifestyles have reached alarming levels. This condition is associated with early clustering of cardiovascular risk factors, including hypertension, dyslipidemia, hyperuricemia, glucose intolerance, and type 2 diabetes. In this population, initial management should prioritize lifestyle and dietary modification rather than pharmacological intervention, except in carefully selected high-risk cases [71].

From a clinical perspective, integrating statin therapy with structured nutritional counseling may optimize LDL-C reduction, improve treatment adherence by avoiding unnecessary dose escalation, and mitigate adverse effects—such as myalgia and liver enzyme abnormalities—that frequently lead to therapy discontinuation [72]. This combined approach also facilitates the management of low-grade inflammation, insulin resistance, and hepatic steatosis, all of which interact with lipid metabolism and can influence responsiveness to statin therapy.

In summary, while clinical guidelines provide a robust methodological foundation for appropriate statin prescribing, they should not be applied uncritically. Their greatest clinical effectiveness is achieved when recommendations are tailored to the individual patient and when pharmacological treatment is integrated with dietary and lifestyle interventions.

## 5. Link Between Malnutrition and Statin Therapy

Long-term polypharmacy is associated with anorexia and gastrointestinal impairment and may negatively affect nutritional status by altering taste perception, nutrient absorption, metabolism, and the balance of essential vitamins and minerals [24]. Conversely, malnutrition reduces drug bioavailability and alters pharmacokinetic and pharmacodynamic responses, increasing sensitivity to medications and the risk of adverse effects at standard doses. This bidirectional relationship creates a vicious cycle in which polypharmacy worsens nutritional status, further amplifying drug-related toxicity [73]. These considerations highlight the importance of assessing nutritional status before prescribing medications.

Recent evidence has highlighted that the use of statin monotherapy in the primary prevention of cardiovascular disease among patients with metabolic syndrome is associated with suboptimal diet quality [74]. The link between malnutrition, or states of nutritional depletion, and an increased risk of statin side effects is clinically relevant yet frequently under-recognized.

Malnourished patients represent a fragile biological milieu, in which the metabolic effects of statins, especially at the hepatic and muscular levels, may become less tolerable and potentially more harmful. Individuals with a low body mass index (BMI) and/or protein–calorie malnutrition have reduced total muscle mass and, consequently, a smaller volume of drug distribution. In such patients, a standard statin dose that would be appropriate for an individual of normal body weight may be relatively excessive, thereby increasing the risk of toxic effects [75]. Malnutrition, particularly when associated with advanced liver disease or chronic alcohol consumption, may also impair cytochrome P450 (CYP450) enzyme activity, which is essential for the metabolism of most statins. This impairment can result in drug accumulation, thereby increasing the risk of hepatotoxicity [76].

Furthermore, sarcopenia secondary to malnutrition leads to a reduced functional reserve of skeletal muscle and a diminished capacity to adapt to pharmacological stress [77,78]. Given that statins can induce mitochondrial dysfunction and endoplasmic reticulum stress (ER-stress) in muscle fibers, malnourished muscle tissue is inherently more vulnerable to pharmacological insults, including those associated with statin therapy [79]. As previously discussed, statins inhibit the synthesis of mevalonate, a precursor not only of cholesterol but also of CoQ10, which is essential for mitochondrial ATP production and functions as a key antioxidant [80]. Malnourished patients often exhibit reduced baseline CoQ10 levels; statin therapy may therefore exacerbate pre-existing mitochondrial energy deficits and oxidative stress, leading to muscle pain and weakness [81].

Low serum vitamin D concentrations also represent a recognized risk factor for statin-induced myopathy. Vitamin D plays a critical role in skeletal muscle health by regulating mitochondrial function, muscle contractility, and protein synthesis. Malnutrition, particularly when combined with limited sunlight exposure—a common condition in elderly and chronically ill individuals—amplifies vitamin D deficiency, thereby predisposing muscle tissue to statin-related damage [82,83]. However, it should be noted that a recent randomized clinical trial demonstrated that vitamin D supplementation did not prevent SAMSs or reduce statin discontinuation rates [84].

## 6. Nutrition and Statin Therapy

Clinical evidence supporting the use of nutraceuticals to improve lipid levels is highly variable and, for many compounds, still very limited. Data on the use of nutraceuticals in patients with SAMSs or statin intolerance are even more scarce [85]. For most nutraceuticals, a recommendation can be made only based on their demonstrated efficacy in lowering LDL-C and available safety data. Therefore, there is an urgent need for additional evidence on the potential role of nutraceuticals in patients experiencing statin-associated adverse effects. Unfortunately, nearly 50% of individuals initiating statin therapy do not achieve the targeted reduction in plasma LDL-C and thus remain exposed to a substantial residual risk of CVD [86]. Consequently, additional strategies may be useful to improve the efficacy of statin treatment and to limit potential adverse effects.

Statin therapy reduces plasma LDL-C and high-sensitivity C-reactive protein levels, resulting in a significant improvement in CV risk. It has therefore been suggested that reducing chronic inflammation through cholesterol lowering may attenuate vascular damage and ultimately improve CV outcomes [85]. In this context, adequate nutrition represents an essential component of statin therapy.

It has been suggested that a “healthy diet” suitable for CVD may increase the effectiveness of statins by reducing inflammation [70]. Although the concept of a healthy diet remains broad and ambiguous, a Mediterranean-style dietary pattern—characterized by a high intake of fruits, vegetables, whole grains, legumes, nuts, olive oil, and unsaturated fats, together with a reduced intake of saturated fats and simple sugars—appears to act synergistically with statins in reducing CV risk. Indeed, adherence to a Mediterranean diet has been shown to reduce all-cause, coronary artery disease (CAD), and cerebrovascular mortality in patients with CVD, independently of statin use. In the same population, statins reduce CV mortality only when combined with a Mediterranean dietary pattern. These findings suggest that the control of low-grade inflammation, rather than lipid lowering alone, may be a key determinant of mortality reduction in these patients [70].

The role of nutraceuticals in controlling LDL-C levels, particularly in statin-intolerant patients, has been comprehensively addressed in recent reviews [85]. Below we limit ourselves to summarizing the main ones.

An important nutraceutical for cholesterol control is red yeast rice (RYR) which contains monacolin K, a compound structurally like lovastatin. Red yeast rice (RYR) has been used as an alternative to statin therapy in patients with mild to moderate hypercholesterolemia, particularly in those with statin intolerance, and clinical studies suggest that it is generally well tolerated, safe, and effective for the primary prevention of CVD [85]. Although effective in improving blood lipid profiles, in Europe, RYR is considered a drug; therefore, when prescribed as a supplement, the monacolin K content should not exceed 3 mg/day [87,88]. Furthermore, RYR has not been adequately studied in the pediatric population, so young subjects treated with RYR should be carefully monitored [71].

Omega-3 fatty acids may also enhance the benefits of statin therapy. A meta-analysis including eight studies and 803 subjects demonstrated that omega-3 combined with statins is superior to the statin alone in stabilizing coronary plaques and promoting plaque regression and may further reduce the occurrence of CV events [89]. However, RCTs from previously published systematic reviews or meta-analyses of statin use or omega-3 supplementation in PubMed involving 264,516 adults suggested that pravastatin and atorvastatin may be more effective than omega-3 supplementation in reducing the risk of total CVD, CAD, and myocardial infarction [90]. Additionally, a multicenter, randomized, double-blind study compared omega-3 fatty acids (eicosapentaenoic acid and docosahexaenoic acid) with corn oil in a total of 13,078 statin-treated patients with high CV risk, hypertriglyceridemia, and low HDL-C levels. In these statin-treated patients, the addition of omega-3 compared to corn oil and usual care did not result in significant differences in major adverse cardiovascular events. These findings do not support routine omega-3 supplementation for reducing major adverse events in high-risk statin-treated patients [91].

Although the anti-inflammatory and cardioprotective properties of omega-3 fatty acids are well recognized, substantial uncertainty remains regarding their effectiveness in preventing or alleviating SAMSs. To date, there is no consolidated clinical evidence demonstrating that omega-3 prevents or significantly reduces SAMSs. Specifically, there is a lack of randomized clinical trials with myopathy- or SAMS-specific endpoints evaluating whether omega-3 supplementation reduces the incidence, severity, or duration of muscle symptoms in patients who develop statin-induced SAMSs. Conversely, some preclinical findings appear promising. A transcriptional and proteomic study conducted in primary human muscle cell lines exposed to a lipophilic statin (simvastatin) and a hydrophilic statin (rosuvastatin) identified more than 1800 transcripts, and 900 proteins were differentially expressed after statin exposure. Both statins significantly impacted cholesterol biosynthesis, fatty acid metabolism, eicosanoid synthesis, proliferation, and differentiation of muscle cells. Notably, eicosanoids restored biological function, suggesting that the addition of omega-3 fatty acids may be useful in preventing or treating SAMSs [92]. Nevertheless, reviews on statin-induced myopathy conclude that clinical evidence supporting specific nutritional interventions, including omega-3 fatty acids, remains limited [93].

Similarly, a recent randomized controlled trial comparing oral CoQ10 supplementation with a placebo in patients with SAMSs showed no significant benefit in improving myopathy [94]. Indeed, CoQ10 levels are reduced by statin therapy, and although supplementation increases circulating CoQ10, clinical trial evidence on its benefit for SAMSs is mixed, with some meta-analyses reporting modest reductions in muscle pain and weakness, while others show no significant effect on muscle pain or CK levels, so CoQ10 cannot be routinely recommended; however, it may be considered on a case-by-case basis in symptomatic patients [94,95,96].

Berberine is a natural alkaloid with cholesterol-lowering properties that acts by increasing hepatic LDL-C uptake. Its effects are mediated through stabilization of LDL receptor mRNA via the ERK signaling pathway and through modulation of PCSK9 expression, leading to increased LDL receptor availability on hepatocytes [85].

Berberine has been found to exhibit biological activities, including reducing blood sugar, regulation of lipids, and anti-arrhythmic and cardio-protective effects [97]. Meta-analyses of randomized controlled trials show that the administration of berberine or berberine-containing products produces modest but significant reductions in LDL-C in patients with dyslipidemia. In a meta-analysis of 41 RCTs with approximately 4800 participants, berberine-containing products significantly reduced LDL-C compared to controls (MD approximately –15 mg/dL; 95% CI: –21 to –9 mg/dL) in addition to decreasing total cholesterol (TC) and triglycerides (TGs) and increasing HDL-C [98]. Berberine, alone or with other nutraceuticals, can have a modest positive impact on LDL-C lowering compared to statins. These impacts are consistent in many dyslipidemic populations and are often accompanied by improvements in TG and HDL-C as well [98].

Plant stanols and sterols have also been shown to reduce excess LDL-C by inhibiting intestinal cholesterol absorption. Accordingly, a meta-analysis of 15 RCTs in patients on statin therapy showed that the intake of plant sterols in combination with statins may exert a synergistic effect in reducing total cholesterol and LDL-C levels by about 0.30 mmol/L more than statin therapy alone, without significant effects on HDL-C or triglycerides [99,100]. However, the role of stanols and sterols in preventing atherosclerotic CVD remains incompletely established.

The concomitant intake of viscous soluble fibers such as psyllium and β-glucan with statin therapy has been shown to further reduce LDL-C compared with statin monotherapy. Randomized controlled trials and meta-analyses support its use as a complementary dietary strategy in dyslipidemia management [101,102,103,104].

Niacin (vitamin B3) is among the nutraceuticals proposed for LDL-C reduction. While the addition of extended-release niacin/laropiprant to statin therapy improved lipid parameters compared with statin dose-doubling in patients with primary hypercholesterolemia or mixed dyslipidemia [105], robust evidence from randomized trials and meta-analyses has shown no additional benefit on major cardiovascular outcomes. Moreover, niacin–statin combination therapy has been associated with an increased risk of adverse effects, including hepatotoxicity, glycemic dysregulation, and statin-associated muscle symptoms, with rare cases of myopathy and rhabdomyolysis, warranting caution and close safety monitoring [106,107]. Table 1 lists the main nutraceuticals considered useful in controling LDL-C levels [85].

## 7. Potential Role of Protein and Essential Amino Acid (EAAs) Supplementation in Prevention of Myopathy and SAMSs

Statins disrupt the delicate balance of amino acid (AA) metabolism and related pathways, leading to energy deficits, oxidative stress, and protein damage in muscles, which manifest clinically as SAMSs. In addition, statins affect protein degradation pathways (ubiquitin–proteasome system), altering the stability of muscle proteins, especially those with certain *N*-terminal amino acids such as lysine and arginine [113,114]. This suggests that statin therapy, if supported by adequate protein-AA supplementation, can mitigate statin-induced myopathy by reducing protein degradation.

Available evidence suggests that adequate protein intake may help support muscle metabolism by supporting mitochondrial health, enhancing muscle anabolism and function in patients undergoing statin therapy. However, data on its actual ability to reduce statin-associated muscle damage remains limited and inconclusive [18,85].

Early clinical studies demonstrated that soy proteins can positively regulate LDL receptor expression, which is often downregulated by hypercholesterolemia or dietary cholesterol intake in humans [115,116]. However, the use of soy proteins may also present limitations. Chronic and high consumption of soy products has been reported to interfere with thyroid function and fertility, as well as to reduce the absorption of several minerals (including calcium, iron, magnesium, copper, and zinc) due to their high phytic acid content. Additional direct effects on the circulatory system have also been described [101]. In a cross-sectional study evaluating the effects of a soy-based drink compared with cow’s milk in 21 hypercholesterolemic patients who were resistant or intolerant to statin therapy, the combination of soy and cow’s milk significantly reduced plasma total cholesterol and LDL-C levels by approximately 7.5% [117].

Evidence from preclinical studies suggests that, in hypercholesterolemic rats treated with rosuvastatin (Rvs), dietary supplementation with high-quality casein and, to a greater extent, soy protein is associated with improvements in liver and kidney indices, modulation of oxidative stress markers, changes in the hepatic expression of lipid-regulatory genes (*SREBP-1c*, *SREBP-2*, *FAS*, and *ACC-1*), and partial attenuation of histopathological alterations. While these findings point to a possible protective effect against Rvs-associated hepatotoxicity, their clinical relevance remains to be established [118].

The hypothesis that creatine monohydrate supplementation could reduce muscle soreness and muscle damage following eccentric exercise in patients treated with high-dose atorvastatin has also been investigated. These studies showed no significant differences in muscle responses between creatine and placebo groups. Interestingly, a significant correlation was observed between low vitamin D levels and elevated CK concentrations after exercise, suggesting that vitamin D deficiency may exacerbate the effects of statins on skeletal muscle and potentially contribute to the development of SAMSs [119]. Two clinical studies have reported promising results for creatine supplementation in statin-induced myopathy [120,121]. More recently, a preliminary study indicated that low-dose creatine supplementation reduced subjective myopathy symptoms in patients with mild or early SAMSs, without affecting serum CK levels [122]. In addition, experimental studies and limited clinical evidence suggest that L-carnitine may improve some manifestations of statin-induced muscle toxicity; however, robust clinical data remain scarce [93].

Essential amino acids (EAAs), particularly branched-chain amino acids (BCAAs), play a key role in maintaining muscle mass, enhancing mitochondrial biogenesis and function, increasing SIRT1 expression, and improving physical endurance in cardiac and skeletal muscle, both in vitro and in middle-aged mice. These effects were associated with upregulation of genes involved in reactive oxygen species defense and reduced ROS production, but were markedly attenuated in endothelial nitric oxide synthase-deficient mice, indicating that the anti-aging effects of EAAs are mediated through mitochondrial biogenesis and eNOS signaling [123]. Moreover, EAAs supplementation has been shown to inhibit fat synthesis and accumulation [124,125] and to regulate numerous metabolic pathways through their action as metabokines [126]. Extensive evidence supports the efficacy of dietary supplementation with a balanced EAAs mixture in maintaining muscle mass and mitochondrial function across several clinical conditions characterized by impaired energy production [127,128,129]. For example, elderly patients with chronic heart failure (CHF) or chronic obstructive pulmonary disease (COPD) showed improved exercise tolerance after 1–3 months of EAA mixture supplementation (8 g/day). In CHF patients, exercise capacity increased by 18.7–23% on bicycle testing and by 12–22% on the 6 min walk test, accompanied by a 25% reduction in resting plasma lactate levels and a 16% improvement in insulin sensitivity (HOMA index). COPD patients experienced comparable benefits [127]. A study conducted on 41 patients (age 49.5+/−21 y) with a post-traumatic vegetative or minimally conscious state demonstrated that EAAs mixture supplementation significantly improved the cognitive recovery of patients [128]. In a randomized study of 80 hospitalized elderly patients, supplementation with oral EAAs (8 g/day) reduced infection risk by 30% compared with a placebo during the first month of hospitalization. EAA-treated patients also showed higher serum albumin and hemoglobin levels and lower C-reactive protein (PCR) concentrations [129].

**Figure 3:**
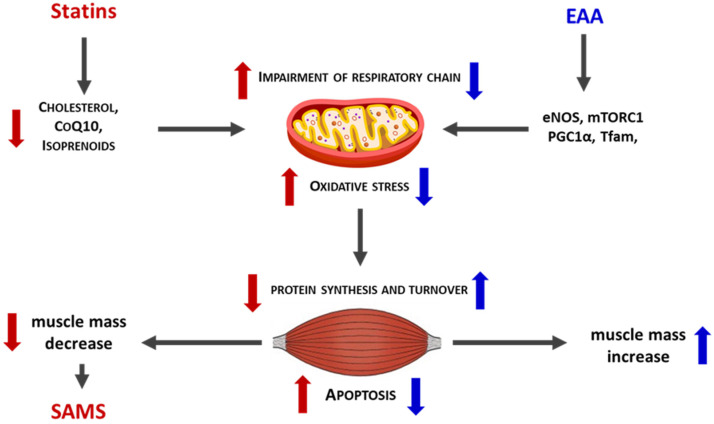
Schematic comparison between the action of statins (red color) and EAAs (blue color) on mitochondria and muscle. EAAs may therefore represent a valid nutritional strategy to limit statin-induced muscle damage and promote muscle metabolism. Thick arrows up = increase. Thick arrows down = decrease.

Numerous preclinical studies have further confirmed the protective effects of EAAs mixtures in hypercatabolic and pathological states, ranging from aging to cancer [130,131,132]. Notably, in preclinical models, a specific balanced EAAs mixture prevented rosuvastatin-induced muscle damage by stimulating de novo protein synthesis and reducing protein degradation. In addition, EAAs supplementation preserved mitochondrial efficiency and improved oxidative stress control in muscle tissue from statin-treated mice [133,134] (Figure 3). Overall, supplementation with a personalized and balanced EAAs mixture may represent a valuable supportive nutritional strategy to complement standard statin therapy, helping to preserve protein and mitochondrial homeostasis and thereby mitigate systemic adverse effects such as lipid accumulation and SAMSs. Accordingly, well-designed clinical trials are warranted to confirm the efficacy of EAAs supplementation, including in patients who are intolerant to statins.

## 8. Conclusions

Although statin therapy remains a valid and effective intervention for lowering LDL-C levels, caution is warranted in the indiscriminate application of clinical guidelines, as well as in the careful monitoring of potential adverse effects. Clinical practice guidelines represent evidence-based tools that support clinical decision-making but do not replace physician judgment. Accordingly, guidelines should be applied within an individualized, patient-centered therapeutic approach that accounts for patient-specific physiological variability and relevant clinical characteristics.

**Figure 4:**
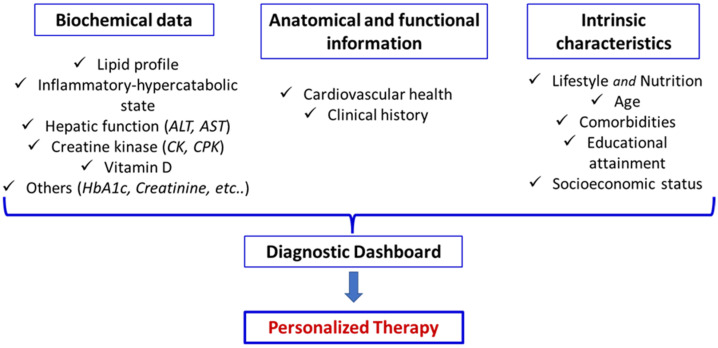
Example of diagnostic dashboard for therapeutic decisions and personalized therapy.

In this context, it is essential to integrate biochemical data of traditional lipid profiles (TC, LDL-C, HDL-C, and TGs), emerging risk biomarkers (e.g., omega-3 index, microRNA levels, triglyceride-to-HDL-C ratio, lipid–lipoprotein ratio), markers of inflammatory and hypercatabolic state (e.g., high-sensitivity C-reactive protein, neutrophile-to-lymphocyte ratio, serum albumin, HOMA-index, and others) and biological parameters of global patients’ metabolism (e.g., complete blood count, HbA1c, liver and kidney functions, creatine kinase, vitamin D, urine tests, and others) together with anatomical and functional assessments of the circulatory system using methods such as carotid ultrasound. These data should be further contextualized within the patient’s clinical history and intrinsic characteristics, including lifestyle, nutrition, age, comorbidities, educational level, and socioeconomic status, to construct a personalized diagnostic dashboard (Figure 4).

Such a dashboard enables the development of tailored therapeutic strategies that, while adhering to established clinical guidelines as a fundamental reference, are specifically adapted to the unique characteristics of each patient. Attention should be devoted to nutritional factors, as adequate intake of the full spectrum of EAAs may provide valuable support to pharmacological therapy by helping to mitigate statin-associated adverse effects on the muscles and liver.

## Figures and Tables

**Table 1:** Nutraceuticals that are believed to be useful as adjuvants to statin therapy: clinical benefits and mechanisms of action. TGs = triglycerides.

Nutraceutical	Clinical Benefit as an Add-on to Statins	Mechanism of Action	Main References
Coenzyme Q10 (CoQ10)	No significant reduction in statin-associated muscle symptoms (SAMSs); improved tolerability.	Improved mitochondrial function; reduced oxidative stress.	Wei, H. et al., *Ir J Med Sci* 2022; [94] Qu, H. et al., *J Am Heart Assoc* 2018; [95] Banach, M. et al., *Mayo Clin Proc*, 2015; [96]
Omega-3 fatty acids (EPA/DHA)	Reduction in triglycerides; reduction in residual CV risk.	Reduction in hepatic VLDL synthesis; anti-inflammatory and antithrombotic effects.	Fan, H. et al., *Am J Cardiol* 2021; [89] Hoang, T. and Kim J. *Nutrients* 2020; [90] Nicholls, S.J. et al., *JAMA* 2020; [91]
Plant sterols/stanols	Additive LDL-C reduction (≈5–15%).	Competition with intestinal cholesterol absorption.	Scholle, J.M. et al. *J Am Coll Nutr* 2009; [99] Han, S. et al., *Sci Rep* 2016; [100]
Soluble fiber (Psyllium, β-glucan)	Reduction in LDL-C and improvement in glycemic control.	Bile acid binding; increased cholesterol excretion.	Cicero, A.F.G. et al., *Nutrients* 2017; [101] Agrawal, A.R., *Int J Clin Pract* 2007; [102] Brum, J. et al., *Am J Cardiol* 2018; [103] Moreyra, A.E., *Arch Intern Med;* 2005 [104]
Berberine	Reduction in LDL-C, TGs and HbA1c; useful in patients with metabolic syndrome.	Upregulation of LDL receptors; AMPK activation.	Banach, M. et al., *Nutrients* 2018; [85] Cai, Y. et al., *Front Pharmacol* 2021; [97] Hernandez, A.V. et al., *J Diet Suppl* 2024; [102]
Red yeast rice (monacolin K)	Marked reduction in LDL-C.	HMG-CoA reductase inhibition.	Banach, M., et al., *Nutrients* 2018; [85] EFSA Panel, *EFSA Journal*, 2018; [87] Cicero, A.F.G. et al., *Nutrients* 2019; [108]
Niacin (vitamin B3)	TG reduction and HDL-C increase (without additional CV benefit).	Reduction in hepatic VLDL synthesis.	Shah, S. et al., *Int J Clin Pract* 2010; [105]; D’Andrea, E., *JAMA Netw Open* 2019; [106] Zambon, A., *Am J Cardiol* 2014; [107]
Vitamin D	Possible SAMS reduction in deficient subjects (inconsistent evidence).	Improved muscle function; inflammatory modulation.	Hlatky, M.A. et al., *JAMA cardiol* 2023; [84] Banach, M., et al., *Nutrients* 2018; [85]
Policosanol	Modest reduction in LDL-C (inconsistent evidence).	Mechanism not clear.	Cicero, A.F.G. et al., *Curr Atheroscler Rep* 2021; [109] Berthold, H.K. et al., *JAMA*, 2006; [110]
Antioxidants (Vitamin E, polyphenols)	Reduction in oxidative stress; possible endothelial protection.	Scavenging of ROS; improved endothelial function.	Patti, A.M. et al., *Arch Med Sci* 2018; [111] Vita, J.A., *Am J Clin Nutr* 2005; [112]

## Data Availability

The data presented in this publication derive from literature research present in scientific databases and indicated in the bibliography.

## Abbreviations

The following abbreviations are used in this manuscript:

CVD	Cardiovascular disease
CK	Creatine kinase
CoQ10	Coenzyme Q10
EAAs	Essential amino acids
HDL-C	High-density lipoprotein cholesterol
HMG-CoA	3-hydroxy-3-methyglutaryl coenzyme-A
LDL-C	Low-density lipoprotein cholesterol
RCTs	Randomized clinical trials
RYR	Red yeast rice
SAMSs	Statin-associated muscle symptoms
TC	Total cholesterol
TGs	Triglycerides
